# Supplementary material for: Molecular Characterization of Dehydrin in Azraq Saltbush among Related Atriplex Species
Source: BioTech (Basel). 2023 Apr 7;12(2):27. doi: 10.3390/biotech12020027 (PMC10123722; doi:10.3390/biotech12020027)
Supplement: Supplementary file 1 [file biotech-12-00027-s001.zip › biotech-2324023-supplementary.pdf]

BioTech

Molecular Characterization of Dehydrin in Azraq Saltbush among Related *Atriplex* Species

Anas Musallam, Saeid Abu-Romman, Monther T. Sadder

Supplementary Table S1. Primer information used in present study.

#	Primer name	T_m	Sequence 5'-3'
1	A.sp._F08	65.6	GAGGCACCCGTACCCGTGGAG
2	A.sp._R08	51.9	CTCCTTTTTCTCTTCATGATC
3	A.sp._R09/10R	53.9	CTCAGCATCATTCTTAGAGTG
4	A.sp._09F	51.9	CGATCGTGGTATGTTTGATTT
5	A.sp._11R	54.9	GCCAGGGATTTTGTCCTTG
6	A.sp._11F	56.1	CAAGCCGTCGTACGAGG
7	A.sp._12F	58.9	ACGTCTCCGAACCCGTCG

Supplementary Table S2. Pore-lining sequence for DHN protein species.

Accession #	Sequence lining	N-terminus	C-terminus
AFC98463	NYKEAPVPVESTDRGM	Cytosolic	Extracellular
AGZ86543	IEKIHVQDHVYSEPSY	Cytosolic	Extracellular
AYH52682	IKIHVQDHVHSEPSY	Cytosolic	Extracellular
AYH52683	EKIHVQDHVHSEPSY	Cytosolic	Extracellular
AYH52684	PIEKIHVQDHVHSEP	Cytosolic	Extracellular
AYH52685	EKIHVQDHVHSEPSY	Cytosolic	Extracellular
AYH52686	EKIHVQDHVYSEPSY	Cytosolic	Extracellular
AYH52687	EKIHVQDHVHSEPSY	Cytosolic	Extracellular
AYH52688	EKIHVQDHVHSEPSY	Cytosolic	Extracellular
AYH52689	EDAVISGVEKAHVFS	Extracellular	Cytosolic
AYH52690	EAVTVATAEPSVEG	Cytosolic	Extracellular
AYH52691	EKIHVQDHVHSEPSY	Cytosolic	Extracellular
AYH52692	EKIHVQDHVHSEPSY	Cytosolic	Extracellular
AYH52693	EKIHVQDHVHSEPSY	Cytosolic	Extracellular
AYH52694	EKIHVQDHVYSEPSY	Cytosolic	Extracellular
AYH52695	EKIHVQDHVYSEPSY	Cytosolic	Extracellular

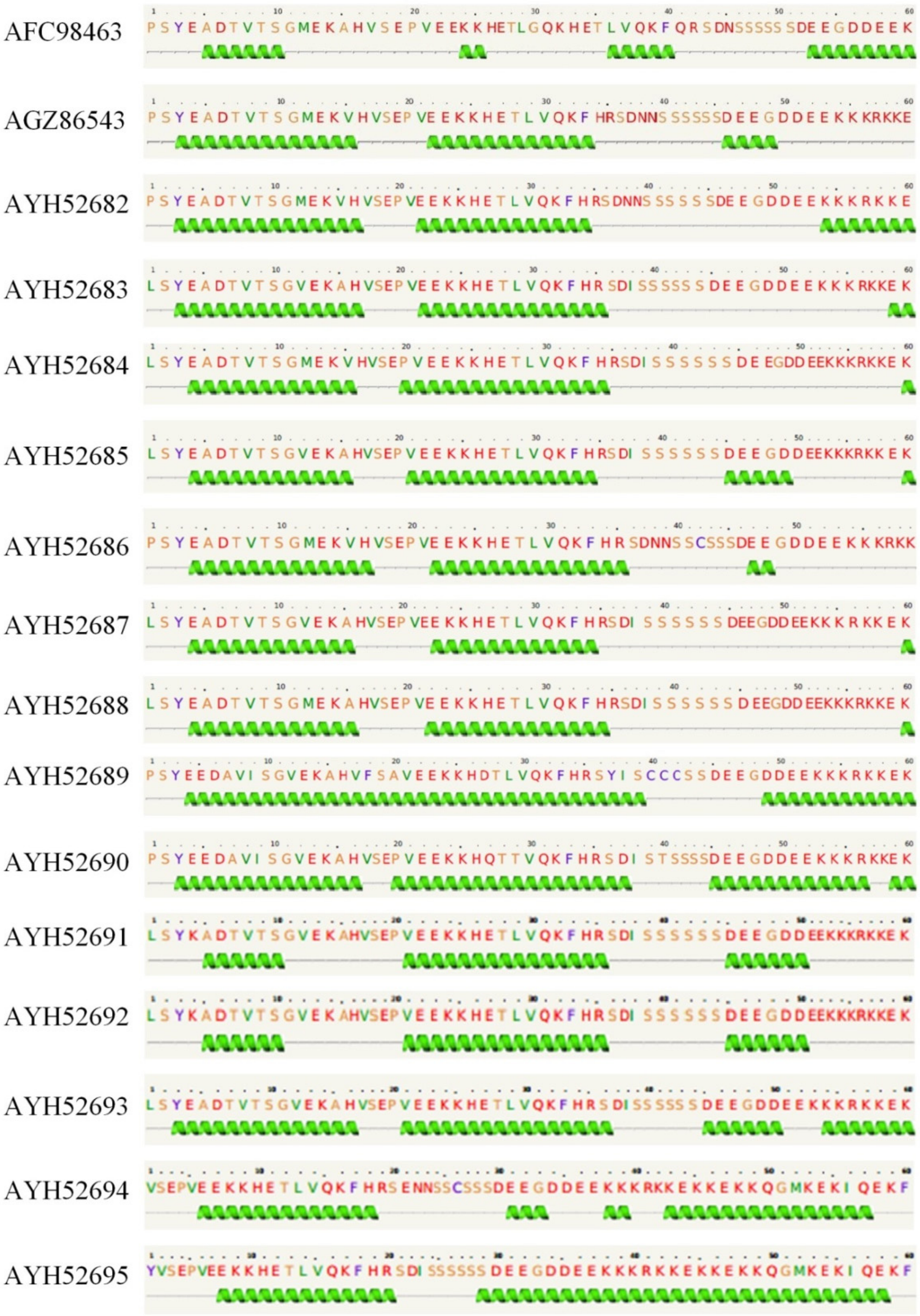

Supplementary Figure S1. *Atriplex* DHN secondary structure prediction spanning N-terminus - S- and K1-segments.
